# A genetic interaction of *NRXN2* with *GABRE*, *SYT1* and *CASK* in migraine patients: a case-control study

**DOI:** 10.1186/s10194-021-01266-y

**Published:** 2021-06-14

**Authors:** Miguel Alves-Ferreira, Marlene Quintas, Jorge Sequeiros, Alda Sousa, José Pereira-Monteiro, Isabel Alonso, João Luís Neto, Carolina Lemos

**Affiliations:** 1 UnIGENe, IBMC - Institute for Molecular and Cell Biology; i3S – Instituto de Investigação e Inovação em Saúde, Universidade do Porto, Porto, Portugal; 2 ICBAS - Instituto Ciências Biomédicas Abel Salazar, Universidade do Porto, Porto, Portugal

**Keywords:** Genetic variants, Neurexin, Synaptic vesicles, Gene-gene interaction, Neurotransmitter

## Abstract

**Background:**

Migraine is a multifactorial disorder that is more frequent (two to four times) in women than in men. In recent years, our research group has focused on the role of neurotransmitter release and its regulation. Neurexin (*NRXN2*) is one of the components of the synaptic vesicle machinery, responsible for connecting intracellular fusion proteins and synaptic vesicles.

Our aim was to continue exploring the role and interaction of proteins involved in the control and promotion of neurotransmission in migraine susceptibility.

**Methods:**

A case-control study was performed comprising 183 migraineurs (148 females and 35 males) and 265 migraine-free controls (202 females and 63 males). Tagging single nucleotide polymorphisms of *NRXN2* were genotyped to assess the association between *NRXN2* and migraine susceptibility. The χ^2^ test was used to compare allele frequencies in cases and controls and odds ratios were estimated with 95% confidence intervals. Haplotype frequencies were compared between groups. Gene-gene interactions were analysed using the Multifactor Dimensionality Reduction v2.0.

**Results:**

We found a statistically significant interaction model (*p* = 0.009) in the female group between the genotypes CG of rs477138 (*NRXN2*) and CT of rs1158605 (*GABRE*). This interaction was validated by logistic regression, showing a significant risk effect [OR = 4.78 (95%CI: 1.76–12.97)] after a Bonferroni correction. Our data also supports a statistically significant interaction model (*p* = 0.011) in the female group between the GG of rs477138 in *NRXN2* and, the rs2244325's GG genotype and rs2998250’s CC genotype of *CASK*. This interaction was also validated by logistic regression, with a protective effect [OR = 0.08 (95%CI: 0.01–0.75)]. A weak interaction model was found between *NRXN2*-*SYT1*. We have not found any statistically significant allelic or haplotypic associations between *NRXN2* and migraine susceptibility.

**Conclusions:**

This study unravels, for the first time, the gene-gene interactions between *NRXN2*, *GABRE -* a GABA_A_-receptor - and *CASK*, importantly it shows the synergetic effect between those genes and its relation with migraine susceptibility.

These gene interactions, which may be a part of a larger network, can potentially help us in better understanding migraine aetiology and in development of new therapeutic approaches.

**Supplementary Information:**

The online version contains supplementary material available at 10.1186/s10194-021-01266-y.

## Background

Although migraine is classified as one of the top 10 most disabling disorders and is the third cause of disability in under 50s, according to the World Health Organization [1, 2] the burden of migraine is still highly underestimated, leading to a high rate of misdiagnosis, mistreatment or even complete absence of treatment [3].

Migraine is a multifactorial disease that results from the small contributions of several genetic and environmental factors and affects more women than men (two to four times) [3]. An increased familial risk for migraine was found in several studies, including in Portuguese families, indicating that migraine has a genetic component [4, 5]. In the last decade, 47 genetic susceptibility *loci* associated with migraine have been reported by genome-wide association studies (GWAS) [6]. Some of the genes present at these *loci* are specifically active in the brain tissues [7]. On the other hand, candidate gene association studies, including in our population, have focused on pathways related to migraine triggers and pathophysiology, namely in genes involved in the vascular and hormonal component and in the release of neurotransmitters [8, 9].

In the world of neurotransmitters’ release, which is undeniably linked to the pathophysiology of this disease, there is a complexity of mechanisms that deserves our attention. Over the past years, we have focused on the synaptic vesicles’ machinery and life-cycle, as our group identified in the Portuguese population two risk variants in the *STX1A* gene [10] coding for syntaxin 1A, a constituent of the SNAP REceptor (SNARE) complex, with a fundamental role in neurotransmission control. Our group also focused in other genes from this pathway with interesting results [9, 11].

Now, we aim to explore this pathway as there are other genes involved that should be taken into account, such as neurexin-2 (*NRXN2*). The neurexin family comprises three neurexin genes (*NRXN1*, *2* and *3*), which differ in their extracellular domains, although no functional differentiation has been determined so far [12]. Neurexin variants play important roles in the central and peripheral nervous system by controlling presynaptic Ca2+ channels at diverse types of synapses (excitatory and inhibitory) as well as the level of their formation and differentiation [13]. Besides binding to synaptotagmin (*SYT*), neurexins bind to GABA_A_-receptors and calcium/calmodulin-dependent serine protein kinase (*CASK)*, but these interactions have not yet been studied in detail [14–16]. Gamma-aminobutyric acid (GABA) is the main inhibitory neurotransmitter in the central nervous system (CNS) [17]. Alterations in GABA_A_-receptors trafficking, function and surface distribution have a crucial role in the modulation of neuronal excitability [18]. In our population, an involvement of *GABA*_*A*_*-R* genes in migraine’s susceptibility was already described [19]. Importantly, several drugs are currently used to prevent migraine through GABA-mediated action [20]. *CASK* is a multidomain scaffolding protein that is involved in synapse targeting, regulation of gene expression [21] and interaction with presynaptic calcium channels [22]. Previously, we have provided data evidencing the role of *CASK* in the pathophysiology of migraine, reinforcing the role of calcium homeostasis in this neurological disorder [11].

Genetic interactions in migraine susceptibility have already been described by others as well as by our group [19, 23, 24]. We consider that the intricacy of migraine does not stem only from the action of a single or several genes, but from an entangled genetic network between them. Therefore, we aimed to further investigate the role and interaction of *NRXN2, SYT1, GABRE* and *CASK*, additional components involved in the control mechanisms of neurotransmitter release apparatus in migraine susceptibility.

## Material & methods

### Aim, subjects and study design

The aim of this study was to investigate the role and interaction of *NRXN2, SYT1, GABRE* and *CASK* in migraine susceptibility. This case-control study was conducted in a sample of patients selected at the outpatient neurologic clinic at Hospital de Santo António (HIS-CHP), Porto. A total of 183 unrelated patients with migraine (148 females and 35 males) were included in the study, after excluding cases with familial hemiplegic migraine. As for controls (202 females and 63 males), they were all migraine-free and either healthy blood donors or women under follow-up at Department of Obstetrics and Gynecology of HIS-CHP. We matched them with cases by geographical origin and age.

Both cases and controls underwent a diagnostic interview, using the same structured questionnaire, based on the operational criteria of the International Headache Society (HIS) [25, 26]. The migraine clinical diagnosis of all patients was later revised by applying the ICHD-II and ICDH-3, but no differences were found (data not shown). Additional information on other clinical diagnosis was collected allowing the exclusion of participants with potential confounding diagnoses. Women with menstrual headaches were excluded from the control group.

The Ethics Committee of HSA-CHP approved the study and participants were asked to give their written informed consent to take part in the study.

### SNPs selection and genotyping

DNA extraction from blood samples were performed by standard salting-out method [27]. Saliva samples were also collected and DNA extraction have been conducted according to the ORAGENE kits manufacturer’s (DNA Genotek) instructions. As shown in Table 1, for *GABRE*, *CASK* and *SYT1* genes we used previous data assessed by our group [9, 11, 19] in the same sample. For *NRXN2,* we selected SNPs from the International HapMap Project data dump (Release 24, November 2008, on NCBI B36 assembly, dbSNP build 126) (www.hapmap.org). Then, using Haploview 4.1 [28], we selected tagging SNPs by an aggressive tagging approach at an r2 threshold of 0.80 and with a minor allele frequency (MAF) of 0.10, capturing all the variation for *NRXN2* gene (Table 1). SNP genotyping was performed using SNaPshot (Applied Biosystems). Product extension primers and SNaPshot single base extension primer sequences for the variants studied can be supplied upon enquiry.

**Table 1:** Tagging SNPs selected for each gene

	Gene			
	***NRXN2***	***GABRE***^**a**^	***CASK***^**b**^	***SYT1***^**c**^
**Tagging SNPs**	rs2269730	rs5970170	rs12857501	rs11113980
	rs3825074	rs1061418	rs57534320	rs3849228
	rs477138	rs2256882	rs5918267	rs7954927
	rs480617	rs1158605	rs5918209	rs17293059
		rs1139916	rs2998250	rs7963801
		rs1003794	rs5918219	rs1245766
		rs5925077	rs5918213	rs10778573
		rs2266858	rs2244325	rs1732664
		rs2266856	rs1150380	rs2037743
			rs5918245	rs2251214
			rs4827286	rs2701566

^a^Quintas M. et al. [19]; ^b^ Quintas M [11]; ^c^ Neto J.L. [9]

The multiplex reactions were performed with the Multiplex PCR Master Mix (Qiagen), in conformity with to the producer’s guidelines. The SNaPshot reaction was executed as per the user manual and genotyped with GeneMapper™ (Applied Biosystems). For rs2269730, genotyping was performed by Sanger sequencing with Big Dye® Terminator Cycle Sequencing 1.1 Ready Reaction (Applied Biosystems) according to standard procedure. The SNaPshot and Sanger sequencing products were loaded in an ABI-PRISM 3130 XL genetic analyser (Applied Biosystems).

### Statistical analysis

We performed a χ^2^ test to compare allele frequencies between the groups of cases and assortment of controls, odds ratios (ORs) were estimated with 95% confidence intervals (CI), using SNPator [29]. We used Haploview 4.1 [28] to compare haplotype frequencies in cases and controls. To identify the protein interaction network with NRXN2, we used protein-protein interaction data retrieved from the STRING database v11 [30]. To obtain only reliable interactions, we considered the human interaction network by selecting only interactions with confidence scores higher than 0.9.

Additionally, we investigated the functional enrichments association related to the protein-protein interactions identified considering Gene Ontologies (GO) [31, 32] for biological processes and cellular components. Multiple comparison significance threshold was set at False Discovery Rate (FDR) < 0.05 [33].

Based on the interactions identified in STRING, we then assessed a possible statistical interaction between variants in these gene sets: *NRXN2*-*SYT1*, *NRXN2*-*GABRE* and *NRXN2*-*CASK* in migraine, using the Multifactor Dimensionality Reduction (MDR) v2.0, a software for the identification of SNP combinations related to disease susceptibility using nonparametric methods and genetic model-free procedures [34]. Permutation Testing Module (version 1.0) of MDR was used to correct for multiple testing, with a 1000-fold permutation test. Taking into account the meaningful and significant gene-gene interactions found by MDR we performed a, multivariable-logistic regression for those variants (considering the most frequent homozygote as the reference) to statistically validate those results. As *GABRE* and *CASK* genes are part of the X chromosome we split the analysis by gender. Bonferroni correction for multiple testing was used and significance was set α = 0.02 in logistic regression analyses performed with IBM SPSS Statistics software (v.24).

## Results

The demographic and clinical information of the participants in the study are presented in Table 2.

**Table 2:** Demographic and clinical data of patients with migraine and controls

Characteristics	Cases (*n* = 183)	Controls (*n* = 265)
Gender, Female/Male	148/35	202/63
Age at observation (mean (SD))	36.14 (12.84)	36.42 (12.35)
Age at onset (mean (SD))	19.76 (11.78)	n/a
Family history of migraine, %	87	n/a

*SD* Standard deviation

### NRXN2

Our first aim was to assess association between *NRXN2* SNPs and migraine susceptibility. Allele frequencies were not significantly different between cases and controls (Additional Table 1). In the global sample, no independent effects of *NRXN2* were found according to multivariate logistic models (Additional Table 2).

Then, a haplotype-based association analysis was also investigated by use of *NRXN2* tagging SNPs. However, no haplotype effect was detected on NRXN2 between cases and controls for any of the haplotypes evaluated (Additional Table 3).

### Interactions involving *NRXN2*

To better characterize the contribution of NRXN2-related genes to migraine variability, we explored potential protein interactions with a confidence score higher than 0.90 and supported by experiments and functional enrichments pathways. *NRXN2-SYT1* and *NRXN2-CASK* showed a STRING interaction score of 0.95 and 0.98, respectively. Despite STRING interaction score *NRXN2-GABRE* is < 0.90 (perhaps because the interaction with neurexin II is through N-ethylmaleimide-sensitive factor (NSF)), we decided to include this interaction in the analyses since a physical and functionally interaction has been reported [14] as well as its role in migraine’s susceptibility [19]. The GO analysis indicated that these protein interactions are mainly involved in neurotransmitter secretion (GO:0007269, FDR q = 4.4 × 10–4), synapse part (GO:0044456, FDR q = 7.81 × 10–6), and transport (GO:0006810, FDR q = 0.012).

### Role of gene interactions in migraine susceptibility

We found 2 strong synergistic interactions, demonstrated by both MDR software and logistic regression analyses, between migraine susceptibility and *NRXN2*-*GABRE* and *NRXN2*-*CASK*.

### NRXN2-GABRE

**Fig. 1:**
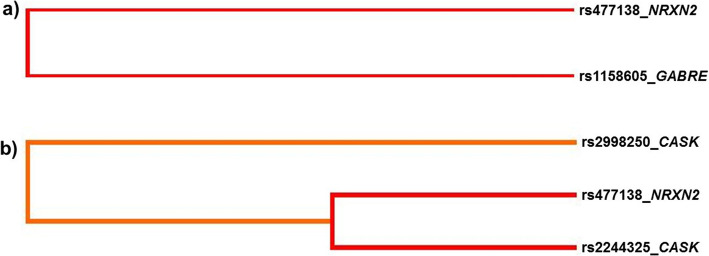
Interaction dendrogram between **a** NRXN2-GABRE and **b** NXRN2-CASK in migraine susceptibility. The colour of the line indicates the type of interaction. Black and dark grey suggest a synergistic relationship. Shorter length of the lines shows a stronger interaction

Importantly, we discovered a substantial synergistic interaction pertaining to the female group - shown in the dendogram (Fig. 1a) - for the best model, between the genotypes CG of rs477138 (*NRXN2*) and CT of rs1158605 (*GABRE*), with a cross-validation consistency (CVC) - the amount of times the model is classified as best within validation sets [35] - was 10/10, with testing balanced accuracy (TBA) of 0.61 and *p* = 0.009 after a test of 1000-fold permutations. This interaction was validated by a logistic regression, showing a significant risk effect [OR = 4.78 (95%CI: 1.76–12.97)] after a Bonferroni correction (Table 3).

**Table 3:** Interaction results found between NRXN2*SYT1; NRXN2*CASK and NRXN2*GABRE, assessed by a logistic regression analysis

Interaction	OR	Migraine CI 95%	***P***-value
NRXN2*SYT1			
rs477138*rs1732664			0.10
CC*TT	1.00	–	–
GC*CT	0.34	0.14–0.82	0.02
NRXN2*CASK			
rs477138*rs2244325*rs2998250			0.30
CC*AA*AA	1.00	–	–
GG*GG*CC	0.08	0.01–0.75	0.03
NRXN2*GABRE			
rs477138*rs1158605			0.001
CC*GG	1.00	–	–
CG*GT	4.78	1.76–12.97	0.002

*NRXN2* Neurexin II, *SYT1* Synaptotagmin 1, *GABRE* Gamma-aminobutyric acid type A receptor epsilon subunit, *OR* Odds ratio, *CI* Confidence interval. (α = 0.02, after Bonferroni correction)

### NRXN2-CASK

In addition, we found a significant interaction in the female group between the GG of rs477138 in *NRXN2* and the GG and CC genotypes of rs2244325 and rs2998250, respectively (*CASK*), with CVC = 10/10, TBA = 0.62 (Fig. 1b), Furthermore, the model remained significant after permutation testing (*p* = 0.011). This interaction was also validated by a logistic regression, with a protective effect [OR = 0.08 (95%CI: 0.01–0.75)] (Table 3). In the male group, we did not find any significant interaction for these gene pairs.

### NRXN2-SYT1

Concerning *NRXN2*-*SYT1* interaction, although MDR showed a weak interaction between these genes (TBA of 0.57; CVC of 9/10, *p* > 0.05 after permutation testing), multivariable-logistic regression indicated a significant possible protective effect [OR = 0.34 (95%CI: 0.14–0.82), *p* = 0.02] (Table 3). However, as these results were not consistent between the two analyses, we did not presume an interaction between the SNPs evaluated in these two genes on migraine susceptibility.

## Discussion

Migraine is a chronic and common neurological condition conceptualized as a complex genetic disorder [3]. This study aimed to investigate the role of genes coding for proteins involved in the control of neurotransmission in migraine susceptibility, thereby improving the knowledge of the mechanisms involved in this phenomenon.

We for the first time show that the genetic interaction and synergetic effect between variants in *NRXN2*, *GABRE* and *CASK*, affects migraine predisposition.

*NRXN2* encodes neurexin II protein, one of three neurexin family members, which play a central role in presynaptic cell adhesion as well as binding intracellular and postsynaptic partners. Interaction in the synaptic cleft of neurexin and neuroligin results in the connection between the two neurons and the production of a synapse [13, 16]. Genetic abnormalities in the neurexin-neuroligin complex leads to an imbalance of excitatory and inhibitory neurotransmission due to loss of presynaptic strength. Deletion of all neurexin family members is lethal and disease-causing variants in neurexin genes has been implicated in the pathomechanisms of schizophrenia and autism spectrum disorders [36].

Until now, no studies have associated this gene with migraine. We did not find any significant association between *NRXN2* and migraine susceptibility in the allelic, genotypic and haplotypic analyses showing that this gene does not act as an independent factor in migraine etiology, in our sample.

### NRXN2 interactors

However, and due to the high complexity of the synaptic vesicle machinery and neurotransmitter release, we decided to focus our analysis on the possible interactors of *NRXN2*. Importantly, we identified two substantial and significant interactions regarding *NRXN2*-*GABRE* and *NRXN2*-*CASK*.

Our analysis showed an increased risk for the female migraineurs that are double heterozygotes for rs477138 (*NRXN2*) and rs1158605 (*GABRE*), when compared with the most common genotypes (Table 3).

*GABRE* is located in chromosome X and belongs to a cluster of GABA receptor genes, encoding for epsilon subunits. Studies carried on in rats show that the subunits are co-expressed in locus cœruleus as well as in other specific brain regions, and may be involved in the migraine [37]. Zhang et al. have shown that presynaptic neurexins physically and functionally interact on postsynaptic GABA_A_-receptors, which may participate in the regulation of the inhibitory/excitatory balance in the brain [14].

Likewise, we also verified the evidence for genetic interaction between *NRXN2* and *CASK* in migraine susceptibility. A significant interaction between rs477138 of *NRXN2* and rs2244325 and rs2998250 of *CASK* was found in the female group, showing a protective effect of the rare homozygotes: GG, CC and GG, respectively (Table 3). The interaction of the CASK PDZ domain with neurexins has been demonstrated [38]; wherein CASK phosphorylates neurexin, although the functional relevance of this activity is still unclear [39]. Alternative splicing neurexin variants were shown to be coupled to synaptic activity via the CASK pathway [16], namely it has been revealed that the activation of the SS#3 splice site in *NRXN2* resulting in exon 11 exclusion depends on depolarization and Ca2+ influx [13]. It is also important to address that disease-causing variants in genes encoding Ca2+ channel subunits result in familial hemiplegic migraine, a monogenic subtype of migraine [40]. Lastly, even though a biological link has been shown between proteins such as *SYT* through the binding to the disordered carboxyterminal domain of neurexins [15], our results do not establish a role for their genetic interaction in migraine susceptibility.

### Gene-gene interactions

Indication of genetic interaction in migraine predisposition has been previously shown, namely, our group had found a robust gene-gene interaction between *BDNF* and *CGRP* and between *GABA*_*A-*_*R* genes, in migraine susceptibility [19, 23]. These studies reinforce the importance of investigating interactions among candidate genes in migraine pathophysiology and also support the genetic interplay in the susceptibility of this disorder.

We performed a logistic regression, including interaction terms considering the *NRXN2*-*GABRE* and *NRXN2*-*CASK* SNPs, in an exploratory-driven analysis of a potential interaction between these gene pairs. The limited number of subjects in each group may impair the estimation of logistic regression parameters thus we chose to perform a MDR analysis. This method combines genotypes into a single dimension with two groups (high or low risk). MDR is also capable of detecting a high-order interaction even in the absence of a main effect with statistical significance. Furthermore, the frequency of false positive results is minimized by the combination of permutation testing and cross-validation [34]. Additionally, MDR has the capability to detect gene-gene interactions even with smaller sample sizes [41], which bestows confidence in our results. All of our interactions were also reaffirmed by a 1000-fold permutation test.

Despite our results being only significant for the female group, differences between genders cannot alone be explained by the influence of X-chromosome genes. Both male and female sex hormones are expected to have a critical impact on the course of the disease. Although the relationship between estrogen and migraine is complex, involving modulation by genomic and non-genomic effects, it is involved, among many other mechanisms, in pain pathways [42]. On the other hand, progesterone appears to have a protective role which might decrease the occurrence of migraine [43]. Likewise, testosterone acts in men as a protective mechanism in the development of pain [43]. These facts may also be responsible for the differential gender ratio found in this disorder but it would be important to assess to what extent our results reflect a true gender-specific effect versus a lower prevalence of migraine in males [44]. However, we should not overlook that practical applications will require more in-depth studies to better understand the pathophysiological mechanisms of migraine.

In conclusion, we found a very significant (two-way) interaction in females between *NRXN2*-*GABRE* and *NRXN2*-*CASK* associated with migraine susceptibility. Based on these results, we hypothesized a convergence of genotypes in these genes associated with migraine susceptibility.

From a clinical point of view, we believe that our study provides important insights in gender-liability differences in migraine. To confirm these results, further studies are needed. Neurogenic mechanisms have been described in previous studies as involved in migraine, including in recent GWAS and also in transgenic mouse models studies [6, 7]. Although GWAS represent a major step in identifying the polygenetic nature of migraine, these large-scale studies typically have a modest impact on interpreting the contribution of any implicated gene to migraine pathophysiology.

## Conclusions

Until now, no studies have aimed at associating the neurexin genes with migraine. The involvement of *NRXN2* gene in migraine is reported for the first time in this study, allowing new hypotheses to be generated that can be translated into new GWA and functional studies to reveal the its underlying biological mechanisms. These gene interactions may be part of a larger network of genes that will help to solve the immense puzzle of the complex etiology of migraine and open the door to novel therapeutic approaches.

## Supplementary Information

**Additional file 1: Additional Table 1.** Allelic association analysis of tagging SNPs selected for NRXN2 gene.

**Additional file 2: Additional Table 2.** Genotypic association of tagging SNPs selected for NRXN2 gene by multivariable logistic regression analysis.

**Additional file 3: Additional Table 3.** Haplotype association analysis of tagging SNPs selected for NRXN2 gene.

## Data Availability

The datasets used and/or analysed during the current study are available from the corresponding author on reasonable request.

## Abbreviations

CASK: Calcium/calmodulin-dependent serine protein kinase
CNS: Central nervous system
CVC: Cross-validation consistency
FDR: False Discovery Rate
GABA: Gamma-aminobutyric acid
GABRE: Gamma-aminobutyric acid type A receptor epsilon subunit
GWAS: Genome-wide association studies
MAF: Minor allele frequency
MDR: Multifactor Dimensionality Reduction
NRXN2: Neurexin
NSF: N-ethylmaleimide-sensitive factor
SNPs: Single nucleotide polymorphisms
SYT: Synaptotagmin
TBA: Testing balanced accuracy
