# Supplementary material for: A genetic interaction of NRXN2 with GABRE, SYT1 and CASK in migraine patients: a case-control study
Source: J Headache Pain. 2021 Jun 14;22(1):57. doi: 10.1186/s10194-021-01266-y (PMC8201896; doi:10.1186/s10194-021-01266-y)
Supplement: Supplementary file 1 — Additional file 1: Additional Table 1. Allelic association analysis of tagging SNPs selected for NRXN2 gene. [file 10194_2021_1266_MOESM1_ESM.docx]

**Additional Table 1:** Allelic association analysis of tagging SNPs selected for *NRXN2* gene.

| SNP | **Alleles (%)** | | **χ2** | **Odds Ratio (95% CI)** | **P-value** |
| --- | --- | --- | --- | --- | --- |
|  | **Cases** | **Controls** |  |  |  |
| rs3825074 |  |  | 0.3 | 1.11 (0.75, 1.67) | 0.58 |
| A | 48 (13.1) | 63 (11.9) |  |  |  |
| G | 318 (86.9) | 467 (88.1) |  |  |  |
| rs2269730 |  |  | 0.09 | 1.06 (0.74, 1.51) | 0.76 |
| A | 60 (16.4) | 91 (17.2) |  |  |  |
| G | 306 (83.6) | 439 (82.8) |  |  |  |
| rs477138 |  |  | 2.96 | 1.30 (0.96, 1.76) | 0.09 |
| C | 261 (71.3) | 405 (76.4) |  |  |  |
| G | 105 (28.7) | 125 (23.6) |  |  |  |
| rs480617 |  |  | 0.92 | 1.17 (0.85,1.61) | 0.34 |
| C | 77 (21.0) | 126 (23.8) |  |  |  |
| T | 289 (79.0) | 404 (76.2) |  |  |  |

SNP, single-nucleotide polymorphism; CI, confidence interval
