# Supplementary material for: A genetic interaction of NRXN2 with GABRE, SYT1 and CASK in migraine patients: a case-control study
Source: J Headache Pain. 2021 Jun 14;22(1):57. doi: 10.1186/s10194-021-01266-y (PMC8201896; doi:10.1186/s10194-021-01266-y)
Supplement: Supplementary file 2 — Additional file 2: Additional Table 2. Genotypic association of tagging SNPs selected for NRXN2 gene by multivariable logistic regression analysis. [file 10194_2021_1266_MOESM2_ESM.docx]

**Additional Table 2:** Genotypic association of tagging SNPs selected for *NRXN2* gene by multivariable logistic regression analysis

| SNP | **Alleles (%)** | | **Odds Ratio (95% CI)** | **P-value** |
| --- | --- | --- | --- | --- |
|  | **Cases** | **Controls** |  |  |
| rs3825074 |  |  |  | 0.26 |
| GG (ref) | 137 (74.9) | 205 (77.4) | 1.00 (-) |  |
| GA | 44 (24.0) | 57 (21.5) | 0.16 (0.01, 3.62) | 0.25 |
| AA | 2 (1.1) | 3 (1.1) | 0.27 (0.01, 5.34) | 0.39 |
| rs2269730 |  |  |  | 0.61 |
| GG (ref) | 126 (68.9) | 181 (68.3) | 1.00 (-) |  |
| GA | 54 (29.5) | 77 (29.1) | 3.18 (0.32, 31.86) | 0.33 |
| AA | 3 (1.6) | 7 (2.6) | 2.89 (0.31, 27.15) | 0.35 |
| rs477138 |  |  |  | 0.25 |
| GG (ref) | 92 (50.3) | 158 (59.6) | 1.00 (-) |  |
| GC | 77 (42.1) | 89 (33.6) | 0.78 (0.36, 1.68) | 0.53 |
| CC | 14 (7.7) | 18 (6.8) | 1.11 (0.51, 2.39) | 0.80 |
| rs480617 |  |  |  | 0.46 |
| CC (ref) | 9 (4.9) | 18 (6.8) | 1.00 (-) |  |
| CT | 59 (32.2) | 90 (34.0) | 0.57 (0.20, 1.57) | 0.27 |
| TT | 115 (62.8) | 157 (59.2) | 0.79 (0.48, 1.30) | 0.26 |

SNP, single-nucleotide polymorphism; CI, confidence interval
