# Supplementary material for: A genetic interaction of NRXN2 with GABRE, SYT1 and CASK in migraine patients: a case-control study
Source: J Headache Pain. 2021 Jun 14;22(1):57. doi: 10.1186/s10194-021-01266-y (PMC8201896; doi:10.1186/s10194-021-01266-y)
Supplement: Supplementary file 3 — Additional file 3: Additional Table 3. Haplotype association analysis of tagging SNPs selected for NRXN2 gene. [file 10194_2021_1266_MOESM3_ESM.docx]

**Additional Table 3:** Haplotype association analysis of tagging SNPs selected for *NRXN2* gene.

| ***Haplotype*** | **Haplotype Frequency** | **Haplotype Frequency** | | **P-value** |
| --- | --- | --- | --- | --- |
|  |  | **Case** | **Control** |  |
| *GGCT* | 0.455 | 0.445 | 0.462 | 0.62 |
| *GGGT* | 0.247 | 0.276 | 0.227 | 0.10 |
| *GGCC* | 0.108 | 0.089 | 0.122 | 0.12 |
| *AACC* | 0.104 | 0.107 | 0.101 | 0.80 |
| *GACT* | 0.056 | 0.047 | 0.062 | 0.34 |
| *AGCT* | 0.012 | 0.016 | 0.009 | 0.36 |
